# Performance of risk scores in predicting mortality at 3, 6, and 12 months in patients diagnosed with community-acquired pneumonia

**DOI:** 10.1186/s12890-024-03121-7

**Published:** 2024-07-10

**Authors:** Eduardo Tuta-Quintero, Alirio R. Bastidas, Gabriela Guerrón-Gómez, Isabella Perna-Reyes, Daniela Torres, Laura Garcia, Javier Villanueva, Camilo Acuña, Eathan Mikler, Juan Arcila, Nicolas Chavez, Allison Riviera, Valentina Maldonado, María Galindo, María Fernández, Carolina Schloss, Luis Felipe Reyes

**Affiliations:** 1 School of Medicine, Universidad de La Sabana. Chía, Km 7, Autonorte de Bogota, Chía, 250001 Cundinamarca Colombia; 2 Master’s Student in Epidemiology, Universidad de La Sabana, Chía, Colombia; 3 Unisabana Center for Translational Science, Universidad de La Sabana, Chía, Colombia; 4 Clinica Universidad de La Sabana, Chía, Colombia; 5 Pandemic Sciences Institute, University of Oxford, Oxford, UK

**Keywords:** Pneumonia, Risk score, Mortality, Observational study

## Abstract

**Background:**

Risk scores (RS) evaluate the likelihood of short-term mortality in patients diagnosed with community-acquired pneumonia (CAP). However, there is a scarcity of evidence to determine the risk of long-term mortality. This article aims to compare the effectiveness of 16 scores in predicting mortality at three, six, and twelve months in adult patients with CAP.

**Methods:**

A retrospective cohort study on individuals diagnosed with CAP was conducted across two hospitals in Colombia. Receiver Operating Characteristic (ROC) curves were constructed at 3, 6, and 12 months to assess the predictive ability of death for the following scoring systems: CURB-65, CRB-65, SCAP, CORB, ADROP, NEWS, Pneumonia Shock, REA-ICU, PSI, SMART-COP, SMRT-CO, SOAR, qSOFA, SIRS, CAPSI, and Charlson Comorbidity Index (CCI).

**Results:**

A total of 3688 patients were included in the final analysis. Mortality at 3, 6, and 12 months was 5.2%, 8.3%, and 16.3% respectively. At 3 months, PSI, CCI, and CRB-65 scores showed ROC curves of 0.74 (95% CI: 0.71–0.77), 0.71 (95% CI: 0.67–0.74), and 0.70 (95% CI: 0.66–0.74). At 6 months, PSI and CCI scores showed performances of 0.74 (95% CI: 0.72–0.77) and 0.72 (95% CI: 0.69–0.74), respectively. Finally at 12 months, all evaluated scores showed poor discriminatory capacity, including PSI, which decreased from acceptable to poor with an ROC curve of 0.64 (95% CI: 0.61–0.66).

**Conclusion:**

When predicting mortality in patients with CAP, at 3 months, PSI, CCI, and CRB-65 showed acceptable predictive performances. At 6 months, only PSI and CCI maintained acceptable levels of accuracy. For the 12-month period, all evaluated scores exhibited very limited discriminatory ability, ranging from poor to almost negligible.

**Supplementary Information:**

The online version contains supplementary material available at 10.1186/s12890-024-03121-7.

## Introduction

In community-acquired pneumonia (CAP), the global occurrence ranges from 1.5 to 14 cases per 1000 person-years, making it the leading cause of death among infectious diseases [1, 2]. In the United States, the incidence is estimated to be between 106 and 164 per 10,000 inhabitants, while in Latin America, it reaches 294 cases per 10,000 inhabitants [1–3]. This incidence is particularly pronounced among individuals over 65 years old, those with comorbidities, and/or immunosuppressed individuals [1–4]. Observational studies report a mortality of CAP at 30 days, 6 months and 1 year after hospital discharge of 4.7% (95% CI 3.4–6.8), 8.6% (95% CI 8.3–11.9), and 14.9% (95% CI 12.4–18.2), respectively [5]. Regarding long-term outcomes, mortality rates from CAP requiring hospitalization were 45.7% in older adults within one year of follow-up [6].

Currently, validated scales are available to stratify pneumonia severity, predict prognosis or survival, and anticipate the need for Intensive Care Unit (ICU) admission [1, 4]. These tools provide valuable guidance for initiating early therapeutic strategies, with the goal of positively impacting outcomes, including mortality rates [1, 2, 7]. The implementation of risk scores (RS) is recommended by clinical practice guidelines and consensus from the American Thoracic Society/Infectious Diseases Society of America (ATS/IDSA) to evaluate prognosis and determine the optimal patient management site for CAP [8, 9]. The most recommended RS are the Pneumonia Severity Index (PSI) and confusion, uremia, respiratory rate, BP, and age ≥ 65 years (CURB-65) [10]. However, the clinical applicability of these scores primarily lies in estimating mortality within the initial 30 days, with insufficient evidence for their long-term predictive capacity [10, 11]. Therefore, efforts have been made to determine the long-term predictive capacity of clinical variables in widely studied CAP scores, some of these are estimated glomerular filtration rate, altered mental status, and pleural effusion on chest X-rays, all associated with long-term mortality [12–14].

E. Lubart et al. [15]. investigated 180 patients in a 1-year period, divided into two groups: those with CAP and acute kidney injury (34.4%) and those with CAP and no acute kidney injury (65.6%). They found a significant association between glomerular filtration rate and mortality (chi-square = 37.1, df = 3, *p* < 0.001). However, it was a single- center retrospective study with small sample size and enrolled only elderly patients (SD: 87.9). Also, Garcia-Vidal et al. [16]. analyzed a total of 2457 hospitalized patients with CAP in a prospective, longitudinal and observational study. They found that 41.1% of patients with altered mental status upon admission died within the first 48 h, and only 10.8% survived beyond 30 days. Utilizing RS that encompass intrinsic patient traits, clinical indicators and lab results could identify those at elevated risk of long-term mortality from lower respiratory tract infections [11, 13]. Yet, evidence concerning the utility of clinical variables and RS in CAP patients remains sparse, given the limitations of long-term mortality studies [11–14]. The most commonly applied long-term scale is the Community-Acquired Pneumonia Severity Index (CAPSI). This index developed a predictive model to estimate one-year mortality, with a weighted score that includes: age over 80 years (4 points), congestive heart failure (2 points), dementia (6 points), respiratory rate ≥ 30 breaths per minute (2 points), and blood urea nitrogen over 30 mg/dL (3 points) as predictors of higher risk, achieving a ROC curve of 0.76 [4]. Understanding the long-term performance of these scales is crucial due to the rise in one-year mortality and the lack of information on risk estimation for short-term to one-year variables. This aids in identify impactful scales for effective patient follow-up and mortality prevention, based on the performance in predicting mortality. Hence, this article aims to compare the effectiveness of 16 scores in forecasting mortality at three, six, and twelve months in adult CAP patients.

## Methods

A retrospective cohort study on individuals diagnosed with CAP was conducted across two hospitals in Colombia. Patients were assessed and admitted to emergency room and ICU from January 2010 to January 2020.

### Eligibility criteria

Participants of both sexes, aged 18 years or older, diagnosed with CAP who had spent at least 6 h in the emergency room, or whom had been admitted to the ICU due to this same condition, were recruited. Pneumonia was defined as pulmonary infiltrate on chest X-ray not seen previously, plus at least one symptom compatible with pneumonia such as cough, fever, dyspnea, and/or chest pain [4, 8, 9]. Patients with nosocomial infection, incomplete clinical history, absence of 12-month survival information, and survival of less than 30 days were excluded (thus evaluating exclusively the mortality of hospitalized patients with CAP who survived the first 30 days).

**Fig. 1:**
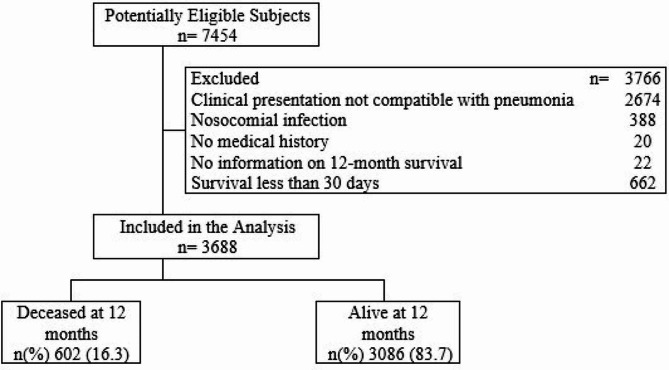
Patient Admission Flowchart

Skilled healthcare professionals, including doctors and specialized nurses, conducted the subject selection process upon entry into the study center. Initially, 7454 potentially eligible subjects were identified, of which 3688 were included in the analysis after excluding those with clinical presentations incompatible with pneumonia, nosocomial infection, lack of medical history, absence of data on 12-month survival, and survival of less than 30 days (Fig. 1).

Additionally, medical records needed to include sufficient information for the evaluation of CURB-65, CRB-65, Severe Community Acquired Pneumonia (SCAP), CORB, ADROP, National Early Warning Score (NEWS), Pneumonia Shock, Risk of Early Admission to ICU (REA-ICU), PSI, SMART-COP, SMRT-CO, SOAR, Quick Sequential Organ Failure Assessment (qSOFA), Systemic Inflammatory Response Syndrome (SIRS), CAPSI, and Charlson Comorbidity Index (CCI) scores (Supplementary Table 1.)

### Variables

Sociodemographic variables (age and gender), comorbidities through the CCI, vital signs, consciousness status, chest X-ray findings such as multilobar involvement or pleural effusion reported by a radiologist, and laboratory tests including arterial gases, hematocrit, white blood cell count, blood urea nitrogen, serum sodium, albumin, and blood glucose were included. Additionally, the need for ICU, invasive mechanical ventilation (IMV), and/or vasopressor support were considered. Sepsis was defined as a life-threatening organ dysfunction resulting from an imbalanced host response to infection [17]. Patients with septic shock were identified based on their need for vasopressors to maintain a mean arterial pressure of > 65 mmHg in the clinical setting [18]. The dependent variable was mortality evaluated at 3, 6, and 12 months following CAP diagnosis.

To minimize possible errors in outcome classification, the research team collecting data from clinical records had medical expertise in diagnosing the studied pathology. To reduce typing bias, at least two team members reviewed the information, and in case of inconsistency, a third team member reviewed the information and made the final decision.

### Sample size

Sample size calculation used data from Lim et al. [19], describing a sensitivity of 75% and specificity of 69% for CURB-65, and Fine et al. [20], reporting a sensitivity of 100% and specificity of 52.2% for PSI. Using the formula for paired diagnostic tests, with an expected mortality of 6.1%, 90% power, and statistical significance of 0.05, a minimum of 625 subjects was required.

### Missing data

An imputation analysis addressed missing data, employing weighted mean imputation for quantitative variables and logistic regression for qualitative variables with a loss of less than 10% [21]. Variables with more than 10% data loss were excluded. A comparison between non-imputed and imputed results ensured that imputation did not introduce bias or significantly alter the original data.

### Statistical analysis

Data were entered into REDCap (Research Electronic Data Capture) [22] for subsequent analysis using SPSS 25 software (IBM Corp. IBM SPSS Statistics for Windows, Version 25.0 licensed). Qualitative variables were reported in frequencies and percentages, while quantitative variables were summarized using mean and standard deviation for normally distributed ones and median and interquartile range for non-normally distributed ones. Bivariate analysis between questionnaires and the outcome (alive or dead) was performed using the chi-square test for qualitative variables and Student’s t-test or Mann-Whitney U test for quantitative variables [21]. The sociodemographic variables, clinical variables, laboratory test results, and diagnostic imaging findings obtained from patients’ medical records were used to calculate the scores for each of the RS included in the study (Supplementary Table 1). Scores obtained for each questionnaire were used to calculate a receiver operating characteristic (ROC) curve, sensitivity, specificity, positive predictive value (PPV), negative predictive value (NPV), positive likelihood ratio (LR+), and negative likelihood ratio (LR-), using the established cutoff point for each questionnaire (Supplementary Table 2). A comparison was made between the different ROC curve obtained, using the DeLong test [19], considering a value of *p* < 0.05 as significant. The ROC curve was interpreted as follows: 0.50, absence of discriminatory capacity; 0.51 to 0.60, almost null discriminatory capacity; 0.61 to 0.69, poor discriminatory ability; > 0.7 to 0.8, acceptable discrimination ability; > 0.8 to 0.9, excellent discriminatory capacity; and > 0.9, outstanding discriminatory capacity [21].

## Results

### Population characteristics

A total of 3688 were included in the final analysis (Fig. 1). Mortality at 3, 6, and 12 months was 5.2%, 8.3%, and 16.3%, respectively. The average age was 63.5 years (SD: 21.39), and 59.3% (2188/3688) of the patients were male. The most common symptoms in the overall population were cough in 82.6% (3045/3688), dyspnea in 67.4% (2486/3688), and fever in 47.6% (1756/3688) (Table 1). The most prevalent comorbidities were arterial hypertension in 46.1% (1699/3688), and COPD in 25.5% (941/3688).

**Table 1:** General characteristics of the population

	Total population *n* = 3688	Alive *n* = 3086	Deaths *n* = 602*
Age in years, mean (SD)	63.5 (21.39)	62 (21.78)	70.7 (17.53)
Males, *n* (%)	2188 (59.3)	1801 (58.4)	387 (64.3)
Cough, *n* (%)	3045 (82.6)	2586 (83.8)	459 (76.2)
Dyspnoea, *n* (%)	2486 (67.4)	2095 (67.9)	391 (65)
Fever, *n* (%)	1756 (47.6)	1500 (48.6)	256 (42.5)
pleuritic pain, *n* (%)	954 (25.9)	843 (27.3)	111 (18.4)
Alteration of consciousness, *n* (%)	158 (8.2)	118 (7.1)	40 (15.7)
Wheezing, *n* (%)	829 (22.5)	719 (23.3)	110 (18.3)
FiO2%, mean (SD)	28.5 (12.34)	28.1 (11.59)	30.3 (14.98)
Arterial hypertension, *n* (%)	1699 (46.1)	1386 (44.9)	313 (52.1)
Chronic heart failure, *n* (%)	447 (12.1)	357 (11.6)	90 [15]
Acute myocardial infarction, *n* (%)	168 (4.6)	136 (4.4)	32 (5.3)
Cerebrovascular disease, *n* (%)	484 [7]	251 (6.3)	233 (10.8)
COPD, *n* (%)	941 (25.5)	770 [23]	171 (28.5)
Mellitus diabetes, *n* (%)	423 (11.5)	342 (11.1)	81 (13.5)
Chronic kidney disease, *n* (%)	201 (5.5)	149 (4.8)	52 (8.7)
Cancer, *n* (%)	237 (6.4)	168 (5.4)	69 (11.5)
Asthma, *n* (%)	79 (2.1)	74 (2.4)	5 (0.8)
Immunosuppression, *n* (%)	148 [4]	115 (3.7)	33 (5.5)

Notes: SD: Standard deviation; n: number; FiO2: Fraction of inspired oxygen; COPD: Chronic obstructive pulmonary disease

*Mortality between 3 to 12 months

### Arterial gases and blood tests

The inspired fraction of oxygen in survivors was 28.1% (SD: 11.59) compared to 30.3% (SD: 14.98) in non-survivors. Blood urea nitrogen was 4.7 mg/dl lower in survivors compared to the deceased group (22.5 vs. 27.2). Laboratory test results are described in Supplementary Table 3.

### Treatment during hospitalization

12.1% (73/602) of deceased patients had septic shock compared to 6.4% (198/3086) of surviving patients (Supplementary Table 4). The use of vasopressor support and systemic corticosteroids was 12.3% (78/602) and 31.6% (190/602) in deceased patients, respectively. The need for ICU was 7.6% higher in deceased patients compared to the survivor group (17.6 vs. 10).

### Performance of RS for Mortality at 3, 6, and 12 months

**Fig. 2:**
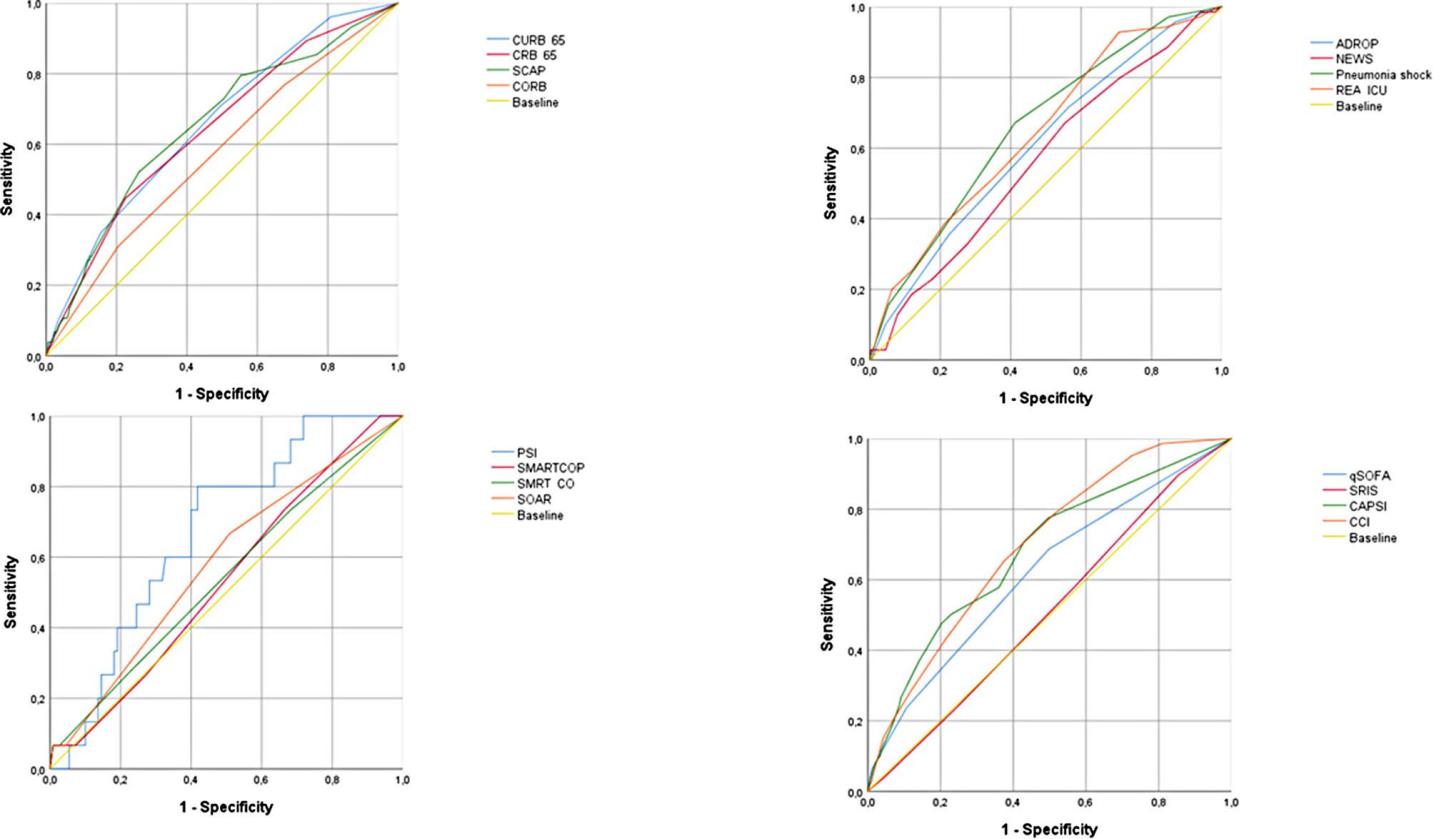
Performance of risk scores at 3-month

**Fig. 3:**
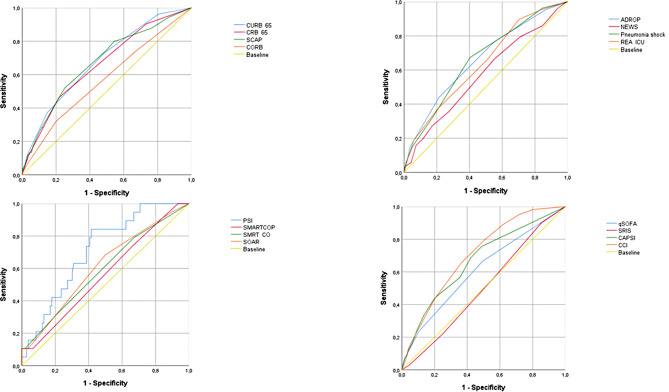
Performance of risk scores at 6-month

**Fig. 4:**
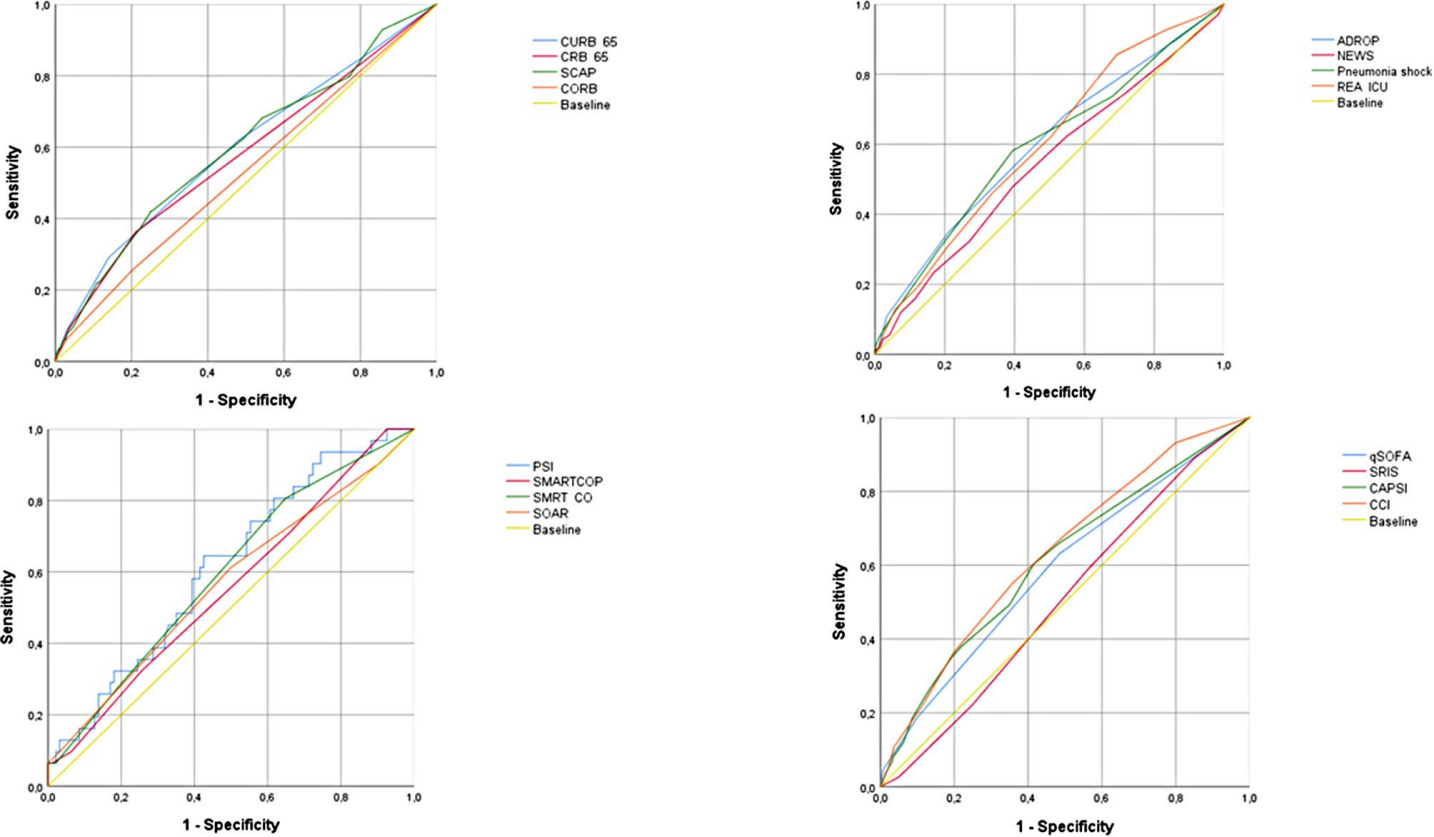
Performance of risk scores at 12-month

At 3 months, PSI, CCI, and CRB-65 scores showed ROC curves of 0.74 (95% CI: 0.71–0.77), 0.71 (95% CI: 0.67–0.74), and 0.70 (95% CI: 0.66–0.74), (Table 2; Fig. 2). At 6 months, PSI and CCI scores showed performances of 0.74 (95% CI: 0.72–0.77) and 0.72 (95% CI: 0.69–0.74), respectively (Table 3; Fig. 3). At 12 months, all evaluated scores showed poor discriminatory capacity, including PSI, which decreased its capacity to poor with an ROC curve of 0.64 (95% CI: 0.61–0.66) (Table 4; Fig. 4). The score with the lowest performance in predicting mortality at 3, 6, and 12 months was SIRS with an ROC curve of 0.51 (95% CI: 0.47–0.55), 0.50 (95% CI: 0.47–0.54), and 0.50 (95% CI: 0.47–0.52), respectively.

**Table 2:** Performance of risk scores in Community Acquired Pneumonia for 3 months Mortality Prediction

	S (CI 95%)	E (CI 95%)	VPP (CI 95%)	VPN (CI 95%)	LR+ (CI 95%)	LR- (CI 95%)	AUCOR (CI 95%)	*p* value*
**Mortality 3 months**								
CURB-65 ≥ 2	71.9 (70.2–73.7)	52.9 (50.9–54.8)	8.7 (7.6–9.8)	96.8 (96.1–97.5)	1.53 (1.283–1.816)	0.53 (0.446–0.632)	0.69 (0.64–0.73)	< 0.001
CRB-65 ≥ 2	49.4 (47.7–51.1)	80.1 (78.7–81.4)	12.3 (11.1–13.4)	96.6 (95.9–97.2)	2.48 (1.836–3.344)	0.63 (0.468–0.853)	0.70 (0.66–0.74)	< 0.001
SCAP ≥ 20	28.6 (26.4–30.7)	23.1 (21.2–25.1)	10.2 (8.8–11.6)	96.2 (95.3–97.1)	1.12 (1.027–1.211)	0.62 (0.568–0.704)	0.66 (0.6–0.72)	< 0.001
CORB ≥ 2	36.3 (34.6–37.9)	81.3 (79.9–82.6)	10.1 (9-11.1)	95.7 (94.9–96.4)	1.93 (1.411–2.653)	0.78 (0.572–1.076)	0.61 (0.56–0.66)	< 0.001
ADROP ≥ 3	44.7 (42.7–46.6)	75.5 (73.8–77.2)	10.5 (9.3–11.7)	95.5 (94.7–96.3)	1.83 (1.373–2.427)	0.73 (0.551–0.974)	0.67 (0.63–0.72)	< 0.001
NEWS ≥ 7	48.2 (46.2–50.1)	64.9 (63-66.8)	5.3 (4.4–6.1)	96.9 (96.2–97.5)	1.37 (1.089–1.73)	0.8 (0.634–1.007)	0.61 (0.56–0.66)	< 0.001
PNEUMONIA SHOCK ≥ 3	70.2 (68.2–72.3)	59.7 (57.5–61.9)	11.2 (9.8–12.6)	96.5 (95.7–97.3)	1.74 (1.412–2.151)	0.5 (0.404–0.616)	0.69 (0.65–0.74)	< 0.001
REA ICU ≥ 7	41.3 (38.8–43.8)	78.1 (76-80.3)	11.3 (9.7–13)	95.2 (94.1–96.3)	1.89 (1.278–2.796)	0.75 (0.508–1.111)	0.65 (0.59–0.7)	< 0.001
PSI ≥ 91	75.3 (72.5–78)	59.7 (56.6–62.8)	9.2 (7.4–11)	97.8 (96.9–98.7)	1.87 (1.374–2.543)	0.41 (0.304–0.563)	0.74 (0.71–0.77)	< 0.001
SMART-COP ≥ 3	60 (58.4–61.6)	52.3 (50.7–53.9)	6.4 (5.6–7.2)	96 (95.4–96.6)	1.26 (1.082–1.464)	0.76 (0.657–0.89)	0.53 (0.39–0.68)	0.679
SMRT-CO ≥ 3	55.9 (54.2–57.6)	66 (64.4–67.6)	8.6 (7.7–9.6)	96.3 (95.7–97)	1.64 (1.336–2.024)	0.67 (0.543–0.822)	0.62 (0.57–0.66)	< 0.001
SOAR ≥ 2	64.3 (62.2–66.5)	54.2 (51.9–56.4)	8.1 (6.9–9.3)	96 (95.2–96.9)	1.4 (1.147–1.717)	0.66 (0.538–0.805)	0.62 (0.57–0.67)	< 0.001
qSOFA ≥ 2	22.1 (20.8–23.4)	90.1 (89.1–91)	10.8 (9.8–11.8)	95.5 (94.8–96.2)	2.23 (1.445–3.436)	0.86 (0.561–1.333)	0.61 (0.57–0.65)	< 0.001
SRIS ≥ 2	57.4 (55.8–59)	44.1 (42.5–45.7)	5.3 (4.6-6)	95 (94.3–95.7)	1.03 (0.901–1.168)	0.97 (0.85–1.101)	0.51 (0.47–0.55)	0.754
CAPSI ≥ 4	57.8 (55.9–59.7)	63.9 (62-65.8)	8.9 (7.8–10)	96.1 (95.4–96.9)	1.6 (1.289–1.992)	1.52 (1.219–1.884)	0.68 (0.63–0.73)	< 0.001
CHARLSON ≥ 3	84.7 (83.6–85.9)	41.7 (40.1–43.3)	7.3 (6.5–8.2)	98.1 (97.6–98.5)	1.45 (1.288–1.641)	0.37 (0.324–0.413)	0.71 (0.67–0.74)	< 0.001

Notes: SE: sensitivity; SP: specificity; PPV: positive predictive value; NPV: negative predictive value; LR: like hood ratio; ROC-curve: Area under the receiver operating characteristic curve

* Comparison of ROC curves utilizing the D-Long test

**Table 3:** Performance of risk scores in Community Acquired Pneumonia for 6 months Mortality Prediction

	S (CI 95%)	E (CI 95%)	VPP (CI 95%)	VPN (CI 95%)	LR+ (CI 95%)	LR- (CI 95%)	AUCOR (CI 95%)	*p* value*
**Mortality 6 months**								
CURB-65 ≥ 2	72.1 (70.4–73.9)	53.8 (51.8–55.7)	13.5 (12.2–14.8)	95.1 (94.2–95.9)	1.56 (1.353-1.8)	0.52 (0.449–0.598)	0.69 (0.66–0.73)	< 0.001
CRB-65 ≥ 2	47 (45.3–48.7)	80.9 (79.5–82.2)	18.8 (17.5–20.2)	94.2 (93.4–95)	2.46 (1.926–3.134)	0.66 (0.514–0.836)	0.69 (0.66–0.72)	< 0.001
SCAP ≥ 20	28.8 (26.6–30.9)	88.4 (86.9–89.9)	20.2 (18.3–22.1)	92.4 (91.2–93.7)	2.49 (1.609–3.843)	0.81 (0.521–1.245)	0.68 (0.63–0.72)	< 0.001
CORB ≥ 2	34.2 (32.5–35.8)	81.7 (80.3–83.1)	15.3 (14-16.5)	92.8 (91.9–93.7)	1.87 (1.447–2.41)	0.81 (0.624–1.039)	0.59 (0.56–0.63)	< 0.001
ADROP ≥ 3	47.2 (45.2–49.1)	76.5 (74.9–78.2)	17.1 (15.6–18.6)	93.4 (92.4–94.4)	2.01 (1.586–2.547)	0.69 (0.545–0.875)	0.68 (0.65–0.71)	< 0.001
NEWS ≥ 7	46.5 (44.6–48.5)	65.2 (63.3–67)	8.3 (7.2–9.4)	94.7 (93.8–95.6)	1.34 (1.107–1.612)	0.82 (0.68–0.99)	0.6 (0.56–0.64)	< 0.001
PNEUMONIA SHOCK ≥ 3	69.3 (67.2–71.3)	60.8 (58.7–63)	17.2 (15.6–18.9)	94.4 (93.4–95.4)	1.77 (1.487–2.105)	0.51 (0.425–0.601)	0.69 (0.65–0.73)	< 0.001
REA ICU ≥ 7	38.2 (35.7–40.7)	82.6 (80.9–84.3)	20.9 (19.1–22.7)	92 (90.8–93.2)	2.24 (1.655–3.041)	0.74 (0.545-1.00)	0.62 (0.58–0.67)	< 0.001
PSI ≥ 91	75.2 (78–78)	60.9 (57.9–64)	14.9 (12.6–17.1)	8.8 (7-10.5)	1.93 (1.492–2.487)	0.41 (0.315–0.524)	0.74 (0.72–0.77)	< 0.001
SMART-COP ≥ 3	62.5 (61-64.1)	53 (51.4–54.6)	10.8 (9.8–11.8)	94 (93.2–94.7)	1.33 (1.178–1.502)	0.71 (0.626–0.799)	0.56 (0.42–0.7)	0.402
SMRT-CO ≥ 3	51.8 (50-53.5)	66.4 (64.8–68)	12.8 (11.6–13.9)	93.5 (92.7–94.4)	1.54 (1.303–1.819)	0.73 (0.615–0.859)	0.59 (0.56–0.63)	< 0.001
SOAR ≥ 2	66.7 (64.6-148700)	55 (52.8–57.2)	12.9 (11.4–14.4)	94.3 (93.3–95.3)	1.48 (1.255–1.751)	0.61 (0.513–0.715)	0.64 (0.6–0.68)	< 0.001
qSOFA ≥ 2	21.5 (20.2–22.8)	90.4 (89.5–91.4)	17 (15.8–18.2)	92.7 (91.9–93.5)	2.25 (1.586–3.194)	0.87 (0.612–1.232)	0.60 (0.57–0.64)	< 0.001
SRIS ≥ 2	57.7 (56.1–59.2)	44.2 (42.6–45.8)	8.6 (7.7–9.5)	92 (91.1–92.9)	1.03 (0.931–1.145)	0.96 (0.865–1.064)	0.50 (0.47–0.54)	0.822
CAPSI ≥ 4	56.5 (54.6–58.4)	64.6 (62.7–66.4)	13.6 (12.3–15)	93.7 (92.8–94.7)	1.59 (1.335–1.905)	1.48 (1.243–1.774)	0.67 (0.63–0.7)	< 0.001
CHARLSON ≥ 3	87.9 (86.9–89)	42.9 (41.3–44.5)	11.4 (10.4–12.4)	97.7 (97.2–98.2)	1.54 (1.397–1.699)	0.28 (0.255–0.31)	0.72 (0.69–0.74)	< 0.001

Notes: SE: sensitivity; SP: specificity; PPV: positive predictive value; NPV: negative predictive value; LR: like hood ratio; ROC-curve: Area under the receiver operating characteristic curve

* Comparison of ROC curves utilizing the D-Long test

**Table 4:** Performance of risk scores in Community Acquired Pneumonia for 12 months Mortality Prediction

	S (CI 95%)	E (CI 95%)	VPP (CI 95%)	VPN (CI 95%)	LR+ (CI 95%)	LR- (CI 95%)	AUCOR (CI 95%)	*p* value*
**Mortality 12 months**								
CURB-65 ≥ 2	63.8 (61.9–65.7)	54.4 (54.4–56.4)	21.6 (20-23.2)	88.4 (87.2–89.7)	1.4 (1.254–1.564)	0.66 (0.595–0.743)	0.63 (0.6–0.66)	< 0.001
CRB-65 ≥ 2	38 (36.3–39.6)	81.5 (81.5–82.9)	27.7 (26.1–29.2)	87.6 (86.5–88.7)	2.06 (1.703–2.482)	0.76 (0.63–0.919)	0.62 (0.59–0.65)	< 0.001
SCAP ≥ 20	22.3 (20.3–24.3)	88.9 (88.9–90.4)	30.7 (28.5–32.9)	83.8 (82.1–85.5)	2 (1.444–2.779)	0.87 (0.63–1.213)	0.60 (0.56–0.63)	< 0.001
CORB ≥ 2	27.7 (26.1–29.3)	81.8 (80.5–83.2)	22.6 (21.1–24)	85.5 (84.3–86.8)	1.52 (1.253–1.853)	0.88 (0.727–1.075)	0.56 (0.53–0.59)	< 0.001
ADROP ≥ 3	38.6 (36.7–40.5)	76.9 (75.3–78.6)	25.2 (23.5–26.9)	86.1 (84.8–87.5)	1.67 (1.393–2.01)	0.8 (0.664–0.959)	0.62 (0.6–0.65)	< 0.001
NEWS ≥ 7	44.7 (42.8–46.6)	65.8 (64-67.7)	14.3 (12.9–15.7)	90.3 (89.2–91.5)	1.31 (1.132–1.512)	0.84 (0.727–0.971)	0.58 (0.55–0.61)	< 0.001
PNEUMONIA SHOCK ≥ 3	58.7 (56.5–60.9)	61.4 (61.4–63.6)	25.7 (23.8–27.7)	86.7 (85.2–88.2)	1.52 (1.328–1.745)	0.67 (0.586–0.77)	0.61 (0.58–0.64)	< 0.001
REA ICU ≥ 7	31.3 (28.9–33.7)	78.8 (76.7–80.9)	25.7 (23.4–27.9)	83.1 (81.1–85)	1.48 (1.158–1.884)	0.87 (0.684–1.112)	0.59 (0.56–0.63)	< 0.001
PSI ≥ 91	57.8 (54.7–60.9)	61 (57.9–64.1)	22.4 (19.8–25.1)	88.1 (86.1–90.2)	1.48 (1.219–1.803)	0.69 (0.569–0.841)	0.64 (0.61–0.66)	< 0.001
SMART-COP ≥ 3	61 (59.4–59.4)	54.1 (52.5–55.8)	20.6 (19.3–21.9)	87.7 (86.6–88.7)	1.33 (1.214–1.457)	0.72 (0.658–0.79)	0.56 (0.44–0.67)	0.352
SMRT-CO ≥ 3	51.7 (49.9–53.4)	67.9 (66.3–69.5)	23.2 (21.7–24.6)	88.2 (87.1–89.3)	1.61 (1.411–1.832)	0.71 (0.625–0.812)	0.61 (0.58–0.63)	< 0.001
SOAR ≥ 2	52.6 (50.3–54.8)	54.3 (52.1–56.5)	20.3 (18.5–22)	83.8 (82.2–85.5)	1.15 (1.017–1.301)	0.87 (0.773–0.988)	0.55 (0.52–0.58)	0.004
qSOFA ≥ 2	17.3 (16.1–18.5)	90.8 (89.8–91.7)	26.7 (25.3–28.2)	84.9 (83.7–86.1)	1.87 (1.439–2.431)	0.91 (0.701–1.184)	0.58 (0.56–0.61)	< 0.001
SRIS ≥ 2	56.8 (55.2–58.4)	44.2 (42.6–45.8)	16.6 (15.4–17.8)	84 (82.8–85.2)	1.02 (0.942–1.099)	0.98 (0.905–1.057)	0.50 (0.47–0.52)	0.790
CAPSI ≥ 4	49.3 (47.3–51.2)	65 (63.2–66.9)	21.7 (20.1–23.3)	86.7 (85.4–88)	1.41 (1.228–1.616)	1.28 (1.117–1.471)	0.61 (0.58–0.64)	< 0.001
CHARLSON ≥ 3	74.1 (72.7–75.5)	43.2 (41.6–44.8)	20.3 (19-21.6)	89.5 (88.5–90.5)	1.3 (1.212–1.402)	0.6 (0.558–0.646)	0.63 (0.6–0.65)	< 0.001

Notes: SE: sensitivity; SP: specificity; PPV: positive predictive value; NPV: negative predictive value; LR: like hood ratio; ROC-curve: Area under the receiver operating characteristic curve

*Comparison of ROC curves utilizing the D-Long test

## Discussion

In this cohort study describing the performance of RS in predicting mortality in patients with CAP, it was observed that at 3 months, PSI, CCI, and CRB-65 showed acceptable predictive performance. At 6 months, only PSI and CCI maintained acceptable levels of accuracy. For the 12-month period, all evaluated scores exhibited very limited discriminatory ability, ranging from poor to nearly negligible. The use of RS may serve as a complementary tool for predicting long-term mortality in hospitalized CAP patients.

Several RS have been proposed to predict the prognosis of severe CAP patients and determine the need for ICU treatment. Anurag et al. [24] described that SCAP (ROC curve: 0.873) performed well in predicting CAP severity compared to PSI (ROC curve: 0.713) and CURB-65 (ROC curve: 0.643). Furthermore, SCAP showed excellent performance in predicting 14-day mortality, with a ROC curve of 0.96. These findings are consistent with those described by España et al. [25], who validated the SCAP score as acceptable for predicting 30-day mortality in CAP patients. However, although studies analyzing SCAP show good performance in predicting short-term mortality [], our study generated new results indicating poor performance of SCAP in predicting mortality at 3 and 6 months, and negligible at 12 months.

Alan et al. [26] described the long-term predictive performance of PSI and CURB-65 over a 6-year follow-up period in hospitalized CAP patients. Initial scores had prognostic accuracy with a ROC curve of 0.79 to 0.83 (*p* < 0.001) and 0.73 to 0.80 (*p* < 0.001) after two years of follow-up, respectively. According to our data, PSI showed the best performance in predicting mortality at 3, 6, and 12 months, while CURB-65 exhibited poor performance in these periods. Additionally, in our comprehensive evaluation of clinical variables and laboratory results, such as comorbidities, renal involvement, and age, CCI and NEWS scores demonstrated a strong negative predictive value independently for long-term mortality from CAP compared to other scores, such as CURB-65, CORB, SMART-COP, among others.

Kaplan et al. [27] assessed 158,960 hospitalized patients, comparing those with CAP to non-CAP controls. Their analysis was one of the first ones to revealed that the most closely associated comorbidities with a higher 1-year mortality rate were metastatic solid tumours (85.5%), renal disease (64.7%), and hepatic disease (56.2%). Currently, most scores for assessing CAP incorporate these comorbidities. However, it’s noteworthy that widely used scores such as CURB-65, CRB-65, SCAP, CORB, ADROP, NEWS, Pneumonia Shock, PSI, SMART-COP, SMRT-CO, SOAR, qSOFA, and SIRS are primarily designed to predict outcomes within a 30-day timeframe. There is a lack of consensus regarding the optimal tool for predicting long-term outcomes, leading to uncertainty when determining mortality risk estimates.

Uranga et al. [4] developed and validated a prognostic index specific for predicting one-year mortality in hospitalized CAP patients. The variables included in the RS construction were age ≥ 80 years with 4 points, chronic heart failure with 2 points, dementia with 6 points, respiratory rate ≥ 30 breaths/min with 2 points, and blood urea nitrogen ≥ 30 mg/dL with 3 points. It was observed that the risk of one-year mortality increased by 24% (HR: 1.24; 95% CI: 1.19–1.28) for each unit increase in the predictive model. The CAPSI showed predictive accuracy of 0.76 in the derivation cohort and 0.77 in the validation cohort, results superior to those reported in our study.

Long-term survival rates exhibit significant variability in the literature. According to Johnstone et al. [28], in their study involving 3284 patients diagnosed with CAP, 12% deceased within the first 30 days, 28% within the first year, and 53% within a 3.8-year follow-up period. The mean age of the deceased was 76.3 years (SD: 13.4), with 56% being male. Conversely, Koskela et al. [29]. described long-term mortality rates in a population with a history of CAP and diabetes, reporting mortality rates of 54% and 10% at 5 years and 11 months of follow-up, respectively, among patients with and without diabetes. Bruns et al. [30]. utilizing municipal records and death certificates in the Netherlands, calculated cumulative mortality rates at 1, 5, and 7 years of 17%, 43%, and 53%, respectively. In comparison with various previous observational studies [23–30], the observed mortality in our study is relatively low, possibly attributable to a younger population, variability in the severity of the illness, and/or the exclusion of subjects with short-term mortality.

### Limitations

Among the limitations of our study is its observational nature, based on information obtained from clinical records, which may have data omissions [21]. It is important to note that the personnel responsible for data collection received adequate training to ensure accuracy in the transcription of the information obtained. Additionally, our study was conducted in two hospital, which may be considered a strength for generalizing the results. By excluding subjects who did not survive the first 30 days, we observed a 15% reduction in the population with CAP, which may influence the overall mortality rate. However, our primary objective is to evaluate the performance of these scores beyond this initial period. We also believe that the sample size included was sufficient to meet the objectives [21].

It is relevant to note that information on causes of death at 12 months could not be obtained, which would have provided important additional data for analysis. Therefore, we suggest that future studies address this issue for a more comprehensive understanding of the results. We consider it pertinent to conduct additional research that can corroborate our findings, as well as externally validate established RS to predict mortality at 12 months, such as those described by Uranga et al. [4], which would help strengthen the evidence and improve accuracy in predicting long-term outcomes in CAP patients.

## Conclusion

In predicting mortality in patients with CAP, it was observed that at 3 months, PSI, CCI, and CRB-65 showed acceptable predictive performances. At 6 months, only PSI and CCI maintained acceptable levels of accuracy. For the 12-month period, all evaluated scores exhibited very limited discriminatory ability, ranging from poor to almost negligible. The use of RS may serve as a complementary tool for predicting long-term mortality in hospitalized CAP patients.

### Electronic supplementary material

Below is the link to the electronic supplementary material.

Supplementary Material 1

## Data Availability

The datasets used and/or analyzed during the current study are available from the corresponding author upon reasonable request.
