# Supplementary material for: Performance of risk scores in predicting mortality at 3, 6, and 12 months in patients diagnosed with community-acquired pneumonia
Source: BMC Pulm Med. 2024 Jul 10;24:334. doi: 10.1186/s12890-024-03121-7 (PMC11238446; doi:10.1186/s12890-024-03121-7)
Supplement: Supplementary file 1 — Supplementary Material 1 [file 12890_2024_3121_MOESM1_ESM.pdf]

Supplementary Table 1. Risk scores used in the study.

CURB-65

Confusion

Uremia >19 mg/ml

Respiratory rate >30 rpm

Blood pressure: Systolic blood pressure < 90 mmHg or dyastolic < 60 mmHg

≥ **65** years

CRB-65

Confusion

Respiratory rate >30 rpm

Blood pressure: Systolic blood pressure < 90 mmHg or dyastolic < 60 mmHg

≥ **65** years

SMART-COP

Systolic blood pressure < 90 mmHg

Multilobar or bilateral infiltrates chest x-ray

Albumin < 3.5 g/dL

Respiratory rate ≥25 bepm if ≤50 years or ≥30 bepm if >50 years

Tachycardia ≥125 bpm

Confusion

Low **O**xygen

≤50 years: pO2 <70 mmHg or SaO2 < 94% or PaO2/FiO2 <333

>50 years: pO2 <60 mmHg or SpO2 < 90% or PaO2/FiO2 <250

PH <7.35

SMRT-CO

Systolic blood pressure < 90 mmHg

Multilobar or bilateral infiltrates chest x-ray

Respiratory rate ≥25 bepm if ≤50 years or ≥30 bepm if >50 years

Tachycardia ≥125 bpm

Confusion

Low **O**xygen

- ≤50 years: pO2 <70 mmHg or SaO2 < 94% or PaO2/FiO2 <333
- >50 years: pO2 <60 mmHg or SpO2 < 90% or PaO2/FiO2 <250

ADROP

Age (Male ≥70 years, female ≥75 years)

Dehydration (Blood urea nitrogen ≥21 mg/dL)

Respiratory failure (arterial oxygen saturation ≤90% or paO2 ≤60 mmHg)

Orientation disturbance

Systolic Blood **P**ressure ≤90 mmHg

Risk of Early Admission to ICU (REA-ICU)

Male

Age < 80 years

Comorbidities

Respiratory rate ≥30 bepm

Heart rate ≥125 bpm

Multilobar infiltrates or pleural effusion

Hypoxemia (SaO2 < 90% or PaO2 < 60 mmHg)

Blood urea nitrogen ≥ 11 mmol/L or ≥ 30 mg/dl

pH < 7.35

Sodium <130 mEq/L

Quick Sequential Organ Failure Assessment (qSOFA)

Systolic blood pressure <100 mmHg

Respiratory rate >20 bepm

Glasgow coma scale ≤14 points

CORB

Confusion

Oxygen saturation ≤90%

Respiratory rate ≥30 bepm

Blood pressure: Systolic blood pressure <90 mmHg or dyastolic ≤60 mmHg

National Early Warning Score (NEWS)

Respiratory rate ≥25 bepm
Oxygen saturation <92%
Requirement of supplementary oxygen
Temperature < 35°c
Systolic blood pressure < 90 or > 140 mmHg
Heart rate <40 or >130 bpm
AVPU (Alert, Voice, Pain, Unresponsive)

Systemic Inflammatory Response Syndrome (SIRS)

Temperature >38°C or <36°C
Heart rate >90 bpm
Respiratory rate >20 bepm or PaCO2 <32 mmHg
White blood cell <4000/mm3 or >12000/mm3

Pneumonia Shock

Age ≥ 75 years
Heart rate ≥ 110 bpm
Hematocrit ≤38%
White blood cell ≥15000/mm3
Sodium ≥ 145 mEq/L
FiO2 ≥ 30%
Requirement of vasopressors
Altered consciousness

Severe Community Acquired Pneumonia (SCAP) Score

Arterial pH <7.30
Systolic blood pressure < 90mmhg
Respiratory rate >30 bepm
Blood urea nitrogen >30mg/dL
Altered consciousness
PaO2<54mmhg or PaO2/FiO2 <250mmhg
Age ≥ 80 years
Multilobar or bilateral infiltrates chest x-ray

Community- acquired pneumonia severity index (CAPSI)

Age > 80 years
Congestive heart failure
Dementia
Heart rate ≥ 30 bpm
Blood urea nitrogen > 30 mg/dL

SOAR

Systolic blood pressure <90 mmHg
Oxygenation PaO2/FiO2 <250
Age ≥65 years
Respiratory rate ≥30 bepm

Charlson comorbidity Index score (CCI)	Pneumonia Severity Index (PSI)
Age	Age
Myocardial infarction	Sex
Congestive heart failure	Nursing home resident
Peripheral vascular disease	Neoplastic disease
cerebrovascular accident or transient ischemic attack	Liver disease history
Dementia	Congestive heart failure history
Chronic pulmonary disease	Cerebrovascular disease history
Connective tissue disease	Renal disease history
Peptic ulcer disease	Altered consciousness
Liver disease	Respiratory rate ≥30 bepm
Diabetes mellitus	Systolic blood pressure <90 mmHg
Hemiplegia	Temperature <35°C (95°F) or >39.9°C (103.8°F)
Moderate to severe Chronic kidney disease	Heart rate ≥125 bpm
Solid tumor	pH <7.35
Leukemia	Blood urea nitrogen ≥30 mg/dL or ≥11 mmol/L
Lymphoma	Sodium<130 mmol/L
Acquired Immunodeficiency Syndrome	Glucose ≥250 mg/dL or ≥14 mmol/L
	Hematocrit <30%
	Partial pressure of oxygen <60 mmHg
	Pleural effusion on X-ray

Notes: mmHg: millimeters of mercury; bepm: breaths per minute; bpm: beats per minute; PaO2: blood oxygen pressure; SaO2: oxygen saturation; FiO2: inspired fraction of oxygen; PaO2: partial pressure of oxygen; PaO2/FiO2: ratio of arterial oxygen partial pressure to fractional inspired oxygen.

Supplementary Table 2. Cohort points in risk scores.

Score	Points	Classification	Management	Cohort points ROC curve
**CURB 65**	0-1	Low risk	Outpatient treatment	≥2
	2	Moderate risk	Hospital admission versus observation admission	
	>/3	High risk	Inpatient treatment	
**PSI**	<51	Class I	Outpatient treatment	≥91%
	51 - 70	Class II	Outpatient treatment	
	71 - 90	Class III	Short hospitalization	
	91 - 130	Class IV	Hospitalization	
	> 131	Class V	Management in ICU	
**SCAP**	Group 0 to 1	Low risk		≥20
	Group 2	Intermediate risk		
	Group 3 to 4	High risk		
**SOAR**	0 to 1	<8% of 30-day mortality risk	Outpatient treatment	≥2
	>/2	33% of 30-day mortality risk	Inpatient treatment	
**CRB 65**	0 to 1	Outpatient treatment		≥2
	2	Short hospitalization		
	3	Hospitalization		
	4	ICU		

**SMART COP**	0 to 2	Low risk of requiring MV and/or VS		≥2
	3 to 4	Moderate risk of requiring MV and/or VS		
	5 to 6	High risk of requiring MV and/or VS		
	7 or higher	Very high risk of requiring MV and/or VS		
**SMRT-CO**	≤ 2	Low risk	ICU admission	≥3
	3	Intermediate risk		
	⩾4	High risk		
**ADROP**	0 to 1	Low risk	Outpatient treatment	≥3
**NEWS**	2	Intermediate risk	Hospitalization	≥7
	3 to 5	High risk	ICU admission	
	0	Low risk	Continue observation	
	1 to 4	Low risk	Continue observation	
	3 in any parameter	Low – moderate risk	Urgent review by a clinician	
	5 to 6	Moderate risk	Urgent review by a clinician	
	7	High risk	Emergency assessment	
**SIRS**	0 to 1	SIRS absent		SIRS present
	2 to 4	SIRS present		
**qSOFA**	>1	Sepsis	10% of mortality risk	≥2
	≤ 3	Class I	1.2% of mortality risk	≥7

**REA ICU**	4 to 6	Class II	6% of mortality risk	
	7 to 8	Class III	9.1% of mortality risk	
	≥ 9	Class IV	15.1% of mortality risk	
**PNEUMONIA SHOCK**	≤2	NS	9.3% of mortality risk	≥3
	≥3	NS	>26% of mortality risk	
**CORB**	0 to 1	Mild	NS	≥2
	2	Moderate	NS	
	3 to 5	Severe	NS	
**Community-acquired pneumonia severity index (CAPSI)**	0-3	Mild	3.35%.4.21%	≥4
	4-5	Moderate	10.33%-12.5%	
	>5	Severe	25%-28.38%	
**Charlson comorbidity Index score**	0	1-year mortality rates	12%	≥ 3
	1-2		26%	
	3-4		52%	
	>5		85%	

Notes: ROC: Receiver Operating Characteristic curve; MV: Mechanical ventilation; VS: Vasopressor support; ICU: Intensive care unit; SIRS: Systemic inflammatory response syndrome; NS: Non specified

Supplementary Table 3. Laboratory tests

	Total population n= 3688	Alive n= 3086	Deaths n= 602*
pH, m(SD)	7.42 (0.06)	7.42 (0.06)	7.43 (0.07)
PaO2, m(SD)	62.1 (19.32)	61.8 (18.09)	63.3 (23.79)
PaCO2, m(SD)	33 (8.32)	33 (8.29)	32.7 (8.45)
HCO3, m(SD)	21 (4.02)	20.9 (3.9)	21.4 (4.45)
BE, m(SD)	88.7 (7.79)	88.7 (7.83)	88.8 (7.63)
Lactate, m(SD)	28.5 (12.34)	28.1 (11.59)	30.3 (14.98)
SpO 2, m(SD)	89.1 (6.7)	89.2 (6.5)	88.9 (7.3)
FiO2 %, mean (SD)	28.5 (12.34)	28.1 (11.59)	30.3 (14.98)
PaO2/FiO2, m(SD)	233 (69.7)	233.8 (69)	229.5 (72.55)
White cells count cell x 10^3, m(SD)	12281.4 (6204.46)	12410.7 (6235.79)	11632.8 (6008.02)
Hemoglobin g/dL, m(SD)	13.5 (2.39)	13.7 (2.27)	13 (2.83)
Hematocrit %, m(SD)	40.4 (6.78)	40.7 (6.55)	38.8 (7.66)
Platelets microliters, m(SD)	259.6 (102.24)	259.4 (100.93)	260.7 (108.52)
Sodium meq/L, m(SD)	137.2 (5.86)	137 (5.44)	138.1 (7.32)
Glucose mg/dL, m(SD)	134.1 (67.66)	133.9 (67.25)	135.2 (69.48)
Albumin mg/dL, m(SD)	3.1 (1.29)	3.3 (0.86)	3.0 (2.12)
Creatinine mg/dL, m(SD)	1.3 (3.2)	1.3 (3.46)	1.4 (1.45)
BUN mg/dL, m(SD)	23.3 (16.83)	22.5 (16.25)	27.2 (18.83)

Notes: SD: Standard deviation; PaO2: Partial pressure of oxygen; PaCO2: Partial Pressure of Carbon Dioxide; HCO_3: Bicarbonate; BE: Base excess; SpO2: Peripheral oxygen saturation; FiO_2: Fraction of inspiratory oxygen concentration; PaO2/FiO_2: The ratio of partial pressure of oxygen in arterial blood to the fraction of inspiratory oxygen concentration; BUN: Blood urea nitrogen.

*Mortality between 3 to 12 months.

Supplementary table 4. Medical treatment and complications.

	Total population n= 3688	Alive n= 3086	Deaths n= 602*
Septic shock, n(%)	271 (7.3)	198 (6.4)	73 (12.1)
Vasopressor support, n(%)	260 (7.3)	182 (6.4)	78 (12.3)
Use of corticosteroid, n(%)	767 (20.8)	577 (18.7)	190 (31.6)
ICU requirement, n(%)	414 (11.2)	308 (10)	106 (17.6)
Days of stay in ICU, m(SD)	11.6 (20.25)	9.6 (11.93)	17.5 (34.47)
IMV, n(%)	266 (7.2)	200 (6.5)	66 (11)
NIMV, n(%)	132 (3.6)	94 (3)	38 (6.3)
Hospitalization requirement, n(%)	3172 (86)	2621 (84.9)	551 (91.5)
Days of stay in Hospitalization, m(SD)	10.7 (83.25)	10.3 (90.71)	12.7 (16.19)

Notes: n: number; SD: Standard deviation; ICU: intensive care unit; IMV: invasive mechanical ventilation; NIMV: Non-invasive mechanical ventilation.

*Mortality between 3 to 12 months.
